# Evaluation of Polyphenolics Content and Antioxidant Activity in Edible Wild Fruits

**DOI:** 10.1155/2019/1381989

**Published:** 2019-01-16

**Authors:** Sharui Shan, Xuming Huang, Munir H. Shah, Arshad Mehmood Abbasi

**Affiliations:** ^1^The First Affiliated Hospital/School of Clinical Medicine of Guangdong Pharmaceutical University, Guangzhou 510006, China; ^2^Department of Chemistry, Quaid-i-Azam University, Islamabad 45320, Pakistan; ^3^School of Light Industry and Food Science, South China University of Technology, Guangzhou 510641, China; ^4^Department of Environmental Sciences, COMSATS University Islamabad (Abbottabad Campus 22060), Pakistan

## Abstract

Plant diversity is a basic source of food and medicines for the local communities of the Himalayas. Current study was intended to assess polyphenolics content and antioxidant potential in edible wild fruits used as food and to treat various diseases by the inhabitants of Himalayan region of Pakistan. The fruits of 20 plant species were evaluated using standard protocols, whereas information on medicinal uses was gathered through semistructured interviews. Comparatively,* Prunus domestica *and* Rubus ellipticus *fruits exhibited highest levels of phenolics and flavonols contents (113.55 ± 0.61 mg GAE/100 g and 200.06 ± 1.57 mg RtE/100 g FW, respectively) in acetone extract. Nevertheless, flavonoids were maximum in the water extract of* Rosa moschata *(194.82 ± 3.46 mg RtE/100 g FW). Contrary,* Duchesnea indica* fruit depicted significant potential to scavenge DPPH and H_2_O_2_ radicals at 94.66 ± 8.89% in acetone extract and 83.54± 9.37% in water extract, while acetone extract of* Rubus ellipticus *had maximum potential to reduce ferric ions (133.66 ± 15.00 *µ*M GAE/100 g FW). Additionally, total antioxidant capacity was highest in the acetone extract of* Berberis lycium *fruit (332.08 ± 21.90 *µ*M AAE/100 g FW). The relationships between polyphenolics and antioxidant activity revealed synergistic role of secondary metabolites in the prevention of diseases. Our study revealed that wild fruits consumed by the local communities of Himalayas are rich in health beneficial phytochemicals and hold significant potential to treat chronic diseases, particularly associated with free radicals.

## 1. Introduction

The use of plants as food and to treat various health disorders is the most pertinent and trustworthy motive to manage the plant diversity [1]. Traditional systems of medicines are based on use of plant species by the rural communities with diverse ethnic beliefs and such system not only provides traditional medicines for health care but also introduces new sources of foods [2]. Although edible wild fruits, vegetables, mushrooms, nuts, and grains are generally consumed as food, different parts of these species have medicinal value to treat health disorders [2–4]. It is well known that ethnopharmacological investigations are crucial to classify plant species that can be designated for their chemical constituents and pharmacological activities [5]. Edible wild food species have significant utilization in famine conditions, even today many agricultural and hunter gathering societies are dependent on wild food resources [6]. These plants possess notable nutraceuticals having health beneficial properties; because of this there is an increasing trend to incorporate wild food plants as popular diet [6]. Certainly, traditional knowledge of wild plant species having food and medicinal value is an important component of folk culture and contributes considerably in the renaissance of traditions [7]. Due to cultural importance and effectiveness and deficiency of contemporary health services, wild foods are much popular among the rural communities around the globe. Wild foods not only support the natural life but also are an imperative source of traditional ecological information [8].

In the present era, consumers are much cautious about diet, wellbeing, and beauty. These facts are exaggerating the concerns over diet and health. Consumers now demand foods that not only fulfill nutritional supplies but also provide additional physiological advantage [9]. Wild food species are much better and healthy alternatives of the processed food products and drugs. Currently, dietetic and phytochemical investigations on wild food species are now mainly focused on their role in the protection and prevention of health disorders related to oxidative stress and malnutrition. Because the excessive production of free radicals in human is the basic cause of cardiovascular disorders, different types of cancer, autoimmune diseases, rheumatism, ageing, and cataracts etc. [1, 10].

Detection of health beneficial secondary metabolites, which act as antioxidants, has been the main focus of investigators working in the area of functional foods and nutraceuticals [9]. An association between consumption of fruits/vegetables and reduced rate of chronic health disorders like cardiac problems, aging, and cancers of respiratory tract, alimentary canal, lungs, bladder, and breast is well recognized [11, 12]. And all of this is credited to the fact that fruits and vegetables are rich source of health beneficial bioactive substances including polyphenolics, vitamins, and minerals [13]. These compounds may act individually or synergistically by various mechanisms to protect human from free radicals and improve the antioxidant potential of plasma subsequent to the inhibition of atherosclerosis [14].

Consumption of edible wild fruits as food and medicine is closely related to health, spiritual, cultural, and socioeconomic aspects of human life. Himalayan region in Pakistan is rich in plant diversity, particularly food and medicinal plant species, which are of low cost, tasty, and readily available and own nutritional and therapeutic potential [4]. Profiling of secondary metabolites and assessment of pharmacological efficacy of wild food resources, that is, fruits, vegetables, mushrooms, and grains, are substantial to introduce functional foods and novel drugs [15]. Although ethnomedicinal uses and cultural aspects of edible wild fruits of this region have been reported before [16], systematic linkage between traditional uses of wild foods in Pakistan and their pharmacological or phytochemical properties has rarely been investigated so far. Therefore, present study was intended with the aim of estimating polyphenolics (phenolics and flavonoids) and* in-vitro* free radicals inhibition of selected edible wild fruits traditionally consumed by the local inhabitants of Himalayan region of Pakistan. This study is an avenue through which we can learn more about the biological activities and can enhance the variety of alternative natural medicines and functional foods.

## 2. Materials and Methods

### 2.1. Ethnomedicinal Data

Data on traditional uses of edible wild fruits to treat health disorders were collected by direct conversation with local inhabitants and using semistructured interviews following the procedure as explained before [17]. However, medicinal application and folk values of these fruits species have already been reported in detail [4].

### 2.2. Sampling and Extraction

Fresh fruits were collected from different localities of Himalayan region of North Pakistan. Samples' identification was done with the help of Flora of Pakistan while scientific and family names were allotted as reported in Angiosperm Phylogeny Group (APG III, 2009) and The Plant List, 2010 (http://www.theplantlist.org). Fresh fruits were dried at room temperature [18] and afterwards, grinded, sieved, and kept in desiccators for further analysis.

Extraction was done as elucidated earlier [19]. Concisely, 1.0 g powdered sample in triplicate was mixed thoroughly with 10 mL of distilled water and centrifuged at 6000 rpm for 15 min. Supernatants were pooled in clean flasks. The whole process was repeated thrice to extract maximum phytochemicals soluble in water and supernatants were pooled. Afterwards, residues were extracted with acetone (1:10 v/v) using same procedure and supernatants were pooled in separate flasks after centrifugation.

### 2.3. Polyphenolics Estimation

Total phenolics content (TPC) was assessed as described previously [20]. In short, 5 mL Folin-Ciocalteu reagent (10-folds) and 4 mL sodium carbonate (7.5%) were added in 1.0 mL of each fruit extract (in triplicate). This blend was kept for 90 min at room temperature before measuring the absorbance at 760 nm. The TPC was presented as mg gallic acid equivalent/100 g fresh weight of sample (mg GAE/100g, FW).

Total flavonoids content (TFC) was estimated as explained formerly [21]. Concisely, 5 mL of each extract (in triplicate) and 0.3 mL of sodium nitrite (5%) were blended thoroughly for 5 minutes and 0.3 mL of aluminium trichloride (10%) was mixed. This mixture was kept for 6 min at room temperature and 2 mL of sodium hydroxide was added to stop the reaction. After dilution (up to 10 mL) with distilled water absorbance was measured at 510 nm. TFC was expressed as mg rutin equivalent per 100 g of fresh sample (mg RtE/100 g).

Total flavonols content (TFlC) was quantified following the protocol of Kumaran & Karunakaran [22]. Briefly, 2.0 mL of aluminium trichloride (2%), 3 mL of sodium acetate (50 g/L), and 2.0 mL of each extract in triplicate were mixed thoroughly. Mixture was kept at 20°C for 2.5 h and absorption was taken using UV-spectrophotometer at 440 nm. TFLC was presented as mg rutin equivalents per 100 g of fresh sample (mg Rt/100g, FW).

### 2.4. Free Radical Scavenging Activity Assessment (FRSAA)

2,2-diphenyl-1-picrylhydrazyl (DPPH), hydroxyl, and hydrogen peroxide (H_2_O_2_) radicals scavenging assays were used to determine free radical scavenging activity in the studied samples.

DPPH assays were performed as reported previously by Chen et al. [23]. Shortly, 2.0 mL of each extract was vortexed vigorously with DPPH solution (5 mL/0.1 mM). This mixture was incubated in the dark for 30 min at room temperature and absorbance was measured against blank at 517 nm. Percentage inhibition of DPPH was calculated by the formula

(1) $$\begin{array}{c} Inhibition\left(\;\%\;\right)=\frac{\left(A_{Blank}-A_{Sample}\right)}{\left(A_{Blank}\right)}\times 100\% \end{array}$$

The hydroxyl (OH^−^) radical scavenging potential of the studied samples was deliberated using the procedure as described before [24]. 2.0 mL of phosphate buffer (pH 7.2/0.2 M), ferrous sulphate (0.04 mL/0.02 M), and 1,10-phenanthroline (1 mL/0.04 M) were gently mixed with 2.0 mL of each extract (in triplicate). Subsequently, 0.1 mL of H_2_O_2_ (7 mM) was added and mixture was kept for 5 min at room temperature. Absorbance was measured at 560 nm and percentage scavenging of hydroxyl radical was calculated using the following equation:

(2) $$\begin{array}{c} SA\left(\;\%\;\right)=\frac{\left(A_{Blank}-A_{Sample}\right)}{\left(A_{Blank}\right)}\times 100 \end{array}$$

The scavenging potential of fruits' extract against hydrogen peroxide (H_2_O_2_) radical was examined using the method of Aiyegoro and Okoh [25]. Briefly, 4 mL of each fruit extract in triplicate was mixed thoroughly with 2.4 mL of H_2_O_2_ solution (4 mM). This mixture was incubated for 10 min at room temperature. Absorbance was taken at 230 nm against blank (extract without H_2_O_2_). The H_2_O_2_ radical inhibition (%) was determined by the following formula:

(3) $$\begin{array}{c} Scavenging.Activity\left(\;\%\;\right)=\frac{\left(A_{Blank}-A_{Sample}\right)}{\left(A_{Blank}\right)}\times 100 \end{array}$$

### 2.5. Evaluation of the Total Antioxidant Capacity (TAC)

TAC was appraised by ferric ion reducing antioxidant power (FRAP) assay and phosphomolybdenum complex assay (PMA) as reported earlier [23].

In FRAP assay, 2.0 mL of each extract in triplicate and potassium ferricyanide (0.1%) were mixed cautiously in phosphate buffer (0.2 M/pH 6.6). Subsequently, this mixture was placed in a water bath at 50°C for 20 min and trichloroacetic acid (2 mL/10%) was added. Supernatant, distilled water, and 0.01% ferric chloride (2 mL of each) were mixed gently. This mixture was kept at room temperature for 20 min before taking absorbance at 700 nm. Final values were expressed as micromole gallic acid equivalent per 100g based on fresh weight of the sample (*µ*M GAE/100g FW).

The PM assay was conducted following the guidelines as reported earlier [26] using ascorbic acid as standard. Concisely, 2.0 mL of each extract was mixed in triplicate with reagents' solution (6.6 mL) containing sulphuric acid (0.6 mol/L), sodium phosphate (28 mol/L), and ammonium molybdate (4 mol/L). This mixture was kept for 90 min at 95°C. After cooling up to room temperature, absorbance was measured at 695 nm. Final values were articulated as micromole ascorbic acid equivalent per 100g based on fresh weight of each sample (*µ*M AAE/100g FW).

### 2.6. Statistical Analysis

Data were presented as mean ± SD for all triplicate analysis and means difference was calculated by Tukey's multiple comparison test. The* P*-values less than 0.05 were considered statistically significant. The SPSS 13.0 (SPSS Inc., Chicago, IL, USA) and STATISTICA software were used for further analysis of the data. All data were reported as mean ± of triplicate analysis.

## 3. Results and Discussion

### 3.1. Ethnomedicinal Uses of the Edible Wild Fruits

Nontimber forests products contribute significantly in the sustainable community development of over 1.6 billion people worldwide. The plant species growing naturally in the forests and nonagricultural lands are the most important substitutes of staple food and revenue for poor societies, particularly in the mountain regions [27]. Naturally growing plants are not only a source of food but are also used as traditional medicine to treat various diseases in human and animals [28]. In the present study edible wild fruits of 20 plant species belonging to 13 botanical families (Table 1) were scrutinized for total phenolics, flavonoids, and flavonols contents and to scavenge free radical species and for antioxidant capacity assessment.

**Figure 1:**
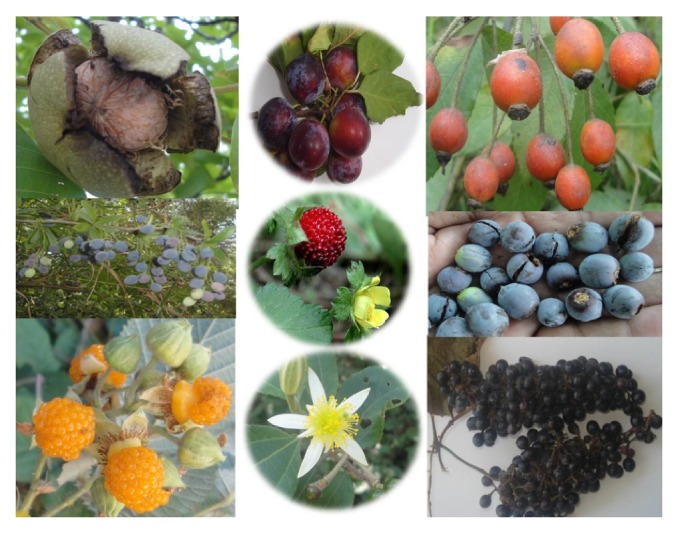
Edible wild fruits of food and medicinal value.

Although other parts such as roots, stem, and leaves of edible wild fruits species are also used to treat various diseases in the region [4, 16, 29, 30], the present study was mainly focused on fruits (Figure 1), which were selected on the basis of ethnomedicinal uses, use reports, availability, and cultural importance index as reported in our previous work [4]. These fruits are commonly used by local inhabitants as food and to treat various diseases, particularly gastrointestinal disorders such as constipation and indigestion [4, 16].* Diospyros lotus, Juglans regia, Myrsine africana, Prunus *spp.,* Rubus *spp., and* Zanthoxylum armatum *fruits are among the commonly utilized in fresh and dried form. Furthermore, fruits of these species also showed quick response in disease treatment. Our findings revealed that, in the study area, traditional phytotherapies are near to decline due to the availability of modern health facilities. To protect the folk belief on medicinal plants, documentation of traditional knowledge of local communities is of utmost importance before its loss forever.

### 3.2. Polyphenolics Content

Ingestion of fresh fruits contributes significantly to the protection and deterrence of diseases [31, 32]. Crude extracts of fruits and vegetables possess powerful antioxidant and anticancer effects, which are mainly accredited to the additive and synergetic effects of phytochemicals [33], such as vitamins, minerals, and polyphenols that provide protection to cellular system against oxidative impairment and consequently reduce the oxidative stress [34]. Pharmacogenetics have suggested that the active constituents should not be purified because in pure or isolated form they may be less active and not perform in the same way as the compounds in whole foods. In the past polyphenolic compounds in fruits and vegetables, particularly tannins, were considered antinutrients because of contrary effects on metabolic rate in human, but recent investigations recognize antioxidative, antimicrobial, anti-inflammatory, hepatoprotective, and anticarcinogenic properties of phenolic compounds and their role in health [35, 36].

**Figure 2:**
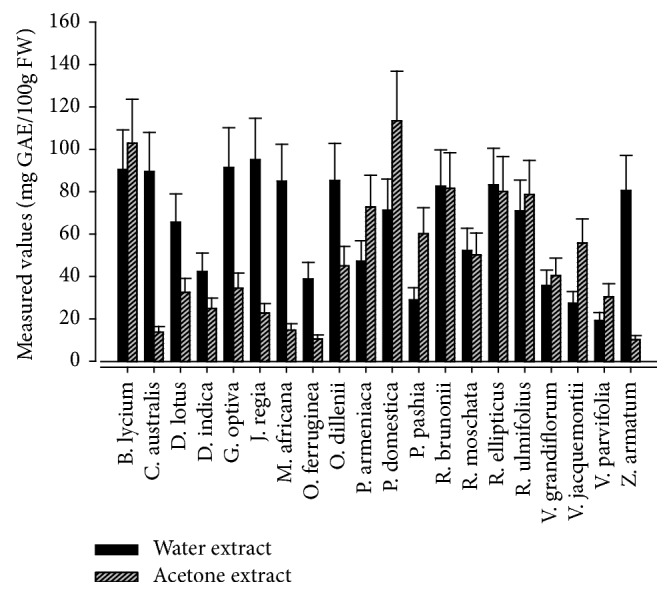
Measured levels of total phenolics content in the edible wild fruits.

Results showing total phenolics content (TPC) determined in the water and acetone extracts of edible wild fruits are presented in Figure 2. On the whole acetone extracts of* Prunus domestica *and* Berberis lycium *depicted highest TPC (113.55 ± 0.61 and 102.9 ± 2.65 mg GAE/100g FW, respectively). However, in majority cases TPC values were higher in water extracts compared to acetone extracts. In the case of water extracts* Juglans regia *fruit contains maximum phenolics content (95.09 ± 0.51 mg GAE/100g FW), followed by* Grewia optiva *and* Berberis lycium *at 91.47 ± 0.86 and 90.57 ± 0.77 mg GAE/100g FW, respectively. It has been reported that* Berberis lycium* fruit possess significant anticarcinogenic, antipyretic, anticoagulant, anti-inflammatory, and hypoglycemic properties [37], which might be attributed to phenolics content and related bioactive constituents. Similarly, fruits and seeds of* Zanthoxylum armatum *are carminative, anthelmintic, and traditionally used as toothache and tonic to treat fever and dyspepsia [38]. Estimated levels of TPC in the fruit of* Zanthoxylum armatum* were comparatively lower than those reported before [39, 40]. However, bioactivities of* Z. armatum* are mainly due to synergistic activities of health beneficial secondary metabolites. Likewise, TPC in the wild edible fruits reported from China and Burkina Faso [41, 42] was significantly higher than what was determined in the present investigation (Figure 2). Contrary, TPC in the water extract of* Rubus ellipticus *was 83.33 ± 0.37 mg GAE/100g FW, which is higher than what was reported earlier 41.08 ± 0.20 mg GAE/100g in the fruit of same species from India [43]. Such variations might be due to change in growing environment, harvesting time, extraction techniques, and genetic difference among varieties. Furthermore, TPC in the edible wild fruits of* Berberis lycium*,* Grewia optiva, Juglans regia, *and* Prunus domestica *examined in the present study was relatively higher than some commonly consumed cultivated fruits, such as pineapple, banana, peach, lemon, orange, and the grape fruit: 94.31, ± 1.54, 90.40 ± 3.22, 84.59 ± 0.71, 81.87 ± 3.50, 81.24 ± 1.10, 70.64 ± 1.58, and 49.60 ± 2.57 mg GAE/100 g, respectively [44]. This confirms that wild food resources, particularly the fruits, are more nutritious and rich in bioactive compounds compared to cultivated fruits, vegetables, and other crops.

**Figure 3:**
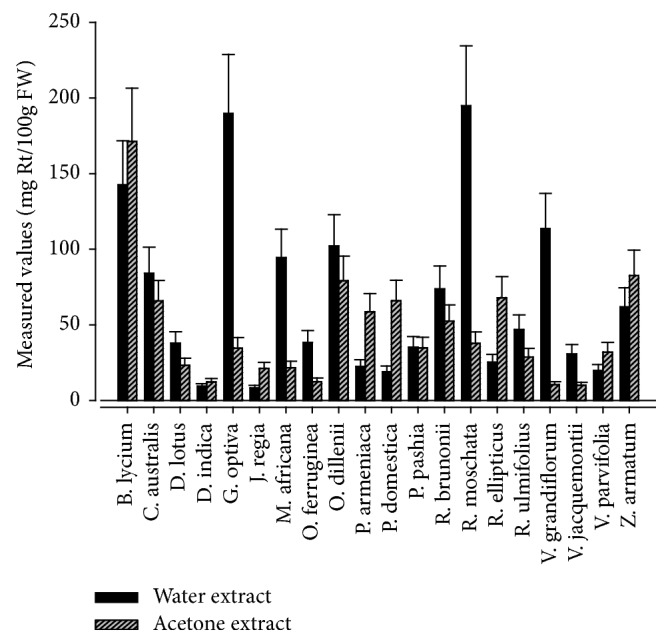
Measured levels of total flavonoids content in the edible wild fruits.

Flavonoids are potent-free radicals scavengers in fruits, vegetables, and medicinal plants. Flavonoids provide protection to lipids and vital cells from oxidative damage [45, 46], involve in the prevention of coronary heart diseases, and exhibit antiproliferative or anticancer activities [45, 47]. Total flavonoids contents (TFC) examined in the edible wild fruits are mentioned in Figure 3. Water extracts of* Rosa moschata, Grewia optiva, Berberis lycium, *and* Viburnum grandiflorum* exhibited maximum TFC: 194.82 ± 3.46, 189.82 ± 1.44, 142.54 ± 1.32, and 113.72 ± 1.50 mg Rt/100 g FW, respectively, with significant difference at* p *< 0.05. In acetone extracts TFC values varied from 10.03 ± 0.52 to 171.31 ± 1.01 mg Rt/100g FW in* Vitis jacquemontii *and* Berberis lycium, *respectively. Measured levels of TFCs determined in the water extracts of* Rubus ellipticus *and* Zanthoxylum armatum* were lower than previous reports [39, 43]. However, calculated values of TFC in the acetone extract of edible wild fruits in the present study were higher than reported for wild fruits of Burkina Faso [42].

**Figure 4:**
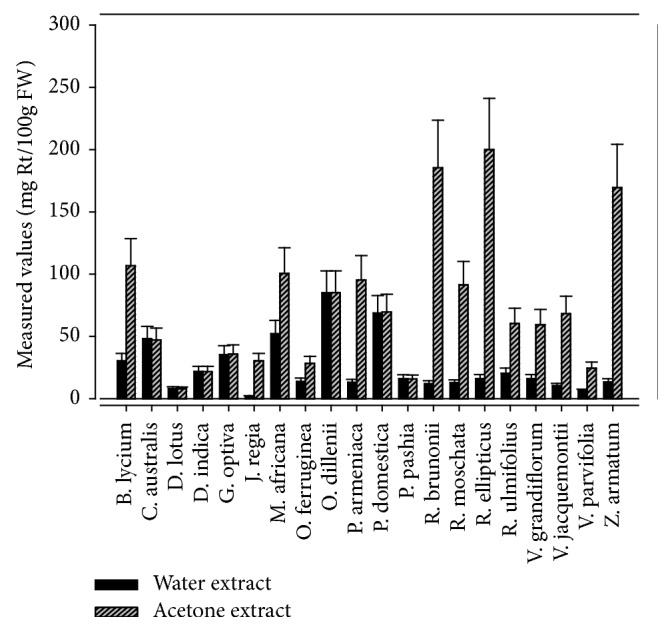
Estimated levels of total flavonols in the edible wild fruits.

Measured values of the total flavonols content (TFlC) in the edible wild fruits of Himalayan region are given in Figure 4. Total flavonols contents in the studied samples were estimated for the first time. Comparatively, in all samples, acetone extracts exhibited more TFlC than water extract. Acetone extract of* Rubus ellipticus *contains highest content of flavonols (200.05 ± 1.57 mg Rt/100g FW), followed by* Rosa brunonii, Zanthoxylum armatum, *and* Berberis lycium *(185.46 ± 0.71, 169.49 ± 1.28, and 106.68 ± 1.34 mg Rt/100g FW, respectively), whereas lowest value was recorded for* Diospyros lotus *(7.91 ± 0.94 mg Rt/100g FW). In the case of water extract* Opuntia dillenii *depicted highest TFlC at 85.08 ± 0.85 mg Rt/100g FW and* Juglans regia *contains lowest value (2.09 ± 0.53 mg Rt/100 g FW). Present study revealed that polyphenolics content in the edible fruits consumed by the inhabitants of Himalayan region of Pakistan was relatively different from previous reports from neighbouring countries. These disparities might be due to genetic variation, difference in species analysed, harvesting season, ripening stage of fruits, growing conditions, and analytical techniques. Genetic and environmental factors contribute significantly to the composition and concentration of secondary metabolites and nutritional quality of fruits and vegetables [48]. It has been reported that reducing compounds other than phenolics such as organic acids and sugars could obstruct the estimation of phenolics [49] and due to low absorption of several flavonoids many other compounds remain underestimated [50].

Majority of the edible wild fruits analyzed are eaten raw and used to treat indigestion, constipation, and other gastrointestinal problems. Consumption of such fruits not only fulfills dietary requirements and health problems, particularly related to digestive system of consumers, but also provides protection in cardiovascular and degenerative diseases, many types of cancer, and aging. Such health benefits are attributed to secondary metabolites including phenolics content and other bioactive constituents. Various epidemiological studies have exposed that adequate ingesting of vegetables, fruits, grains, nuts, and seeds is thought to diminish the jeopardy of several diseases [46].

### 3.3. Free Radicals Scavenging Activities in Edible Wild Fruits

Plant based natural antioxidants have gained the attention by various workers because of dietary and curative properties [51]. Due to complex reactivity of phytochemicals and other natural antioxidants, free radical scavenging power of food and medicinal plants cannot be determined by a single method only. Therefore, use of different assays and at least two test systems is required to authenticate the antioxidant capacity of a plant sample [52]. Consequently, in the present study, free radical scavenging and total antioxidant capacity (TOC) of edible wild fruits were evaluated by DPPH, OH^−^, H_2_O_2_, PMA, and FRAP assays. The scavenging of free radical increases with increasing percentage of their inhibition [53, 54]. Therefore, use of different methods (DPPH, OH^−^, and H_2_O_2_) is helpful to fully explain the antioxidant capacity in plants' extract. Because of different reaction mechanisms, each method only provides approximation of antioxidant capacity [55].

The DPPH radical scavenging assay [56] is the frequently used technique to determine the radical scavenging capacity of plants based extracts [57] because it is a quick and responsive method, which involves simple conventional laboratory equipment. Results for DPPH activity expressed in percentage values are presented in Table 2. Overall, percentage DPPH activity was high in the acetone extract compared to water extracts of the edible wild fruits. In acetone extracts % DPPH ranged from 94.66 ± 8.89 to 54.24 ± 4.69%.* Duchesnea indica, Rubus ellipticus*,* Diospyros lotus, Myrsine africana, *and* Juglans regia *fruits exhibited highly significant DPPH radical scavenging at 94.66 ± 8.89, 94.65 ± 9.87, 94.15 ± 8.75, 93.86 ± 6.98, and 93.35 ± 7.23%, respectively. However, these values were not significantly different at* p *< 0.05. In water extract, % DPPH scavenging activity ranged from 33.66 ± 5.69 to 92.47 ± 5.31%, whereby* Rosa brunonii *fruit had highest % DPPH scavenging and that of* Myrsine africana* showed lowest potential to scavenge DPPH radical (*p *< 0.05). Measured levels of % DPPH activity in the fruits of* Zanthoxylum armatum, Rubus *spp. and* Prunus *spp. were comparable with previous studies [39, 58]. However, in the present study, water and acetone were used as extraction solvent instead of ethanol or methanol. To the best of our knowledge, in other samples % DPPH activity has rarely been reported so far.

OH^−^ is one of the most destructive radicals among all the reactive oxygen species (ROS). This radical reacts with different biomolecules, causing oxidative damage and mutation at cellular level [59, 60]. Present study revealed that percentage OH^−^ radical scavenging activity was comparatively higher in water extracts (Table 2). In water extract, % OH^−^ radical inhibition varied from 75.85 ± 8.31% to 13.15 ± 1.36% (*p* < 0.05). Water extract of* Viburnum grandiflorum *had highest scavenging activity for OH^−^ radical (75.85 ± 8.31%), followed by* Duchesnea indica *and* Pyrus pashia *(75.40 ± 6.11 and 72.37 ± 7.96%, respectively) whereas* Grewia optiva *showed least ability to scavenge OH^−^ radical. Among acetone extracts* Duchesnea indica* showed significant OH^−^ radical scavenging capacity, followed by* Olea ferruginea* and* Grewia optiva *(72.59 ± 6.78, 67.60 ± 7.98, and 63.69 ± 5.59 %, respectively), whereas* Viburnum grandiflorum *fruit exhibited the lowest potential to scavenge OH^−^ radical at 9.840 ± 1.06%. Comparative analysis with reported literature revealed that OH^−^ radical scavenging activity has rarely been reported in all fruit samples except* Zanthoxylum armatum *where in both extracts OH^−^ radical scavenging activity was analogous to previous report [39].

Approximately, 0.28 mg kg^−1^ day^−1^ of H_2_O_2_ enters the human body through breathing, eye, or skin contact [61]. Although H_2_O_2_ is a feeble oxidizing agent, it can deactivate some enzymes. It passes rapidly through cell membrane and reacts with iron II and copper II ions within the cell. This reaction releases OH^−^ radical that initiates lipid peroxidation leading to DNA damage [62]. In the edible fruits of Himalayan region of Pakistan, water extract depicted more activity to scavenge the H_2_O_2_ radical than acetone extract. In water extracts of all samples, H_2_O_2_ radical scavenging activity ranged from 9.51 ± 1.01% to 83.54 ± 9.37% (*p *< 0.05) with maximum value calculated for* Duchesnea indica, *whereas* Grewia optiva *exhibited lowest scavenging power. However, in acetone extract H_2_O_2_ radicals scavenging capacity was highest in* Prunus armeniaca *(73.04 ± 9.21%), followed by* Berberis lycium *(72.87 ± 9.87%) and* Celtis australis *(65.61 ± 8.56%), while lowest value was intended for* Vitis jacquemontii *(2.10 ± 1.00). Comparative analysis of studied samples with reported literature showed that edible fruits of Himalayan region of Pakistan have rarely been investigated before.

### 3.4. Total Antioxidant Capacity (TAC)

The TAC of edible wild fruits was determined using PMA and FRAP assays. In PMA, plant extract is incubated with phosphate-molybdenum (VI), which reduces to green phosphate-molybdenum (V). The reduction of phosphate-molybdenum (VI) is calculated by measuring absorbance at 695 nm [63].

**Figure 5:**
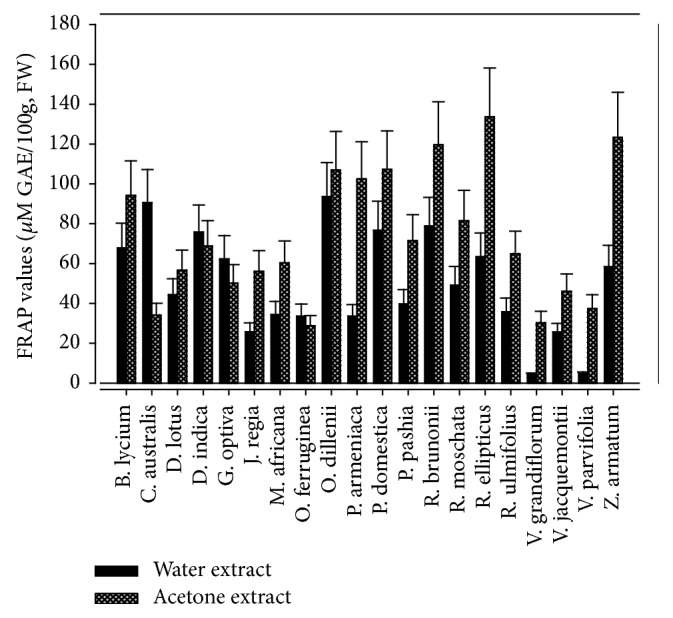
Comparison of FRAP value in water and acetone extracts.

Our results indicate that acetone extracts had maximum TAC (*p *< 0.05) compared to water extract as determined by PM assay (Figure 5). Acetone extract of* Berberis lycium *fruit exhibited highest TAC (332.08 ± 21.91 *µ*M AAE/100g FW), followed by* Rosa brunonii, Zanthoxylum armatum, Rubus ellipticus,* and* Pyrus pashia*: 317.03 ± 23.52, 306.96 ± 24.11, 261.27 ± 17.49, and 223.12 ± 20.09 *µ*M AAE/100g FW, respectively. In the water extract, TAC ranged from 18.36 ± 1.08 to 243.03 ± 21.04 *µ*M AAE/100g FW in* Prunus armeniaca* and* Celtis australis, *respectively. In majority of the samples TAC using PM assay has rarely been reported so far. Although Batool et al. (2010) reported 120.0 *µ*g/mL TAC in the ethanolic extract of* Zanthoxylum armatum *using PM assay, it could not be compared with present findings due to difference in the solvents and extraction methods.

**Figure 6:**
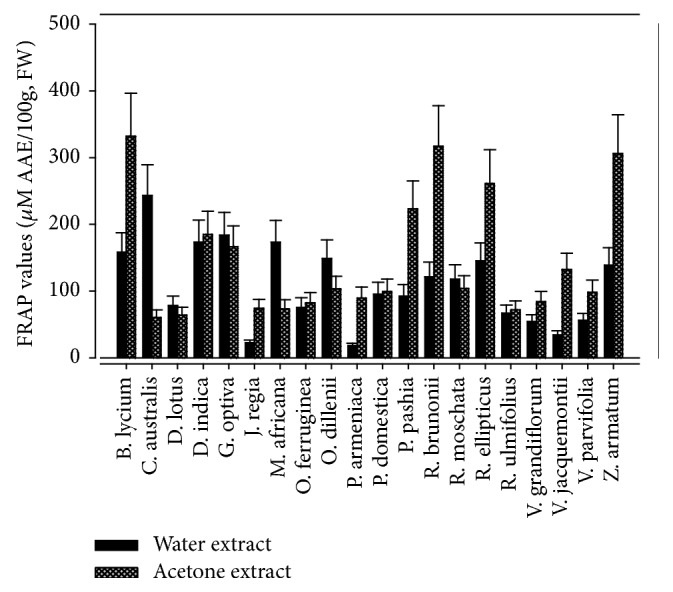
Comparison of PMA value in water and acetone extracts.

Total antioxidative property of foods can also be assessed by computing increase in the absorbance at 595 nm, due to the formation of ferrous ions from FRAP. This increase is associated with combined or “total” reducing power of the phytochemicals in the reaction mixture. The reduction of ferric tripyridyltriazine complex to ferrous can be detected by determining change in absorption at 593 nm [64]. TAC in edible wild fruits determined by FRAP assay is given in Figure 6. Acetone extract of* Rubus ellipticus, Zanthoxylum armatum, Rosa brunonii,* and* Prunus domestica* depicted maximum FRAP values at 133.66 ± 15.03, 123.30 ± 13.77, 119.67 ± 15.41, and 107.33 ± 14.47 *µ*M GAE/100g FW, respectively (*p*<0.05). In the case of water extracts* Opuntia dillenii* fruit had significant FRAP value (93.63 ± 11.10 *µ*M GAE/100g FW), followed by* Celtis australis* and* Rosa brunonii. *Fu et al. [41] reported 57.10 ± 2.06, 1.88 ± 0.13, and 89.60 ± 22.18 *µ*M Fe^2+^g ferric ion reducing antioxidant capacity in the aqueous extracts of wild edible fruits of* Viburnum fordiae, Diospyros kaki, *and* Rosa laevigata. *However, variation in FRAP values are due to genetic difference, extraction method used, type of solvent, and geoclimatic factors.

Although phenolics content and antioxidant properties in the fruits of* Zanthoxylum armatum, Rubus *spp., and* Prunus *spp. have been reported before, phenolics contents and* in vitro* antioxidant properties in other edible wild fruits have never been investigated, particularly with reference to Himalayan region of North Pakistan.

### 3.5. Correlations

Epidemiological studies have proved strong association between phenolics content and antioxidant potential of plant species including fruits, vegetables, and grains [65]. Although numerous antioxidants are involved in the total antioxidant capacity, which constituents are more responsible to scavenge free radicals is not clear yet [55].

Tables 3(a) and 3(b) showed correlations determined between TPC, TFC, TFlC, and antioxidant capacity in edible wild fruits determined by DPPH, OH^−^, H_2_O_2_, FRAP, and PMA assays. In acetone extract TFlC exhibited significant coefficients of determination with total antioxidant properties determined by FRAP and PMA values (r^2^= 0.789 and 0.631), followed by TFC with PMA and FRAP (r^2^= 0.581 and 0.576, respectively). In water extract strong relationships were deliberated for total flavonols and phenolics contents with ferric ion reducing antioxidant potential and PMA value: 0.606, 0.598 and 0.532, respectively. The positive correlation revealed contribution of phenolics content in free radical inhibition and as natural antioxidants [21]. Additionally, such relations demonstrated that the abovementioned antioxidant assays are feasible and correspond to the free radical scavenging activities [66]. However, negative correlations were also calculated between phenolics and DPPH, OH^−^, and H_2_O_2_ free radical scavenging activities. This suggests that phytochemicals other than those studied in the present analysis may be involved in DPPH, OH^−^, and H_2_O_2_ free radical scavenging activities

## 4. Conclusion

Our findings revealed that the studied samples have significant potential to hunt free radicals and are rich in natural antioxidants, particularly phenolics compounds. These fruits are not only used to treat various health disorders, but could also contribute significantly to the prevention of degenerative diseases. In this context, present analysis is the gateway for in-depth and comprehensive phytochemical profiling,* in vivo *biological activities and antioxidant activity and medicinally important wild edible fruits of the region. Furthermore, edible wild fruits species could be an alternative source of income generation for local inhabitants of the area and may be used as germplasm by horticulturists and fruit farmers to introduce new varieties and to provide healthy and more delicious fruits for consumers.

## Figures and Tables

**Table 1:** Medicinal uses of selected edible wild fruits.

**Botanical name**	**Vernacular/English name **	**Family**	**Medicinal use**
*Berberis lycium* Royle	Sumbal/Barberry	Berberidaceae	Tonic, weakness, constipation
*Celtis australis *L.	Batker/European hackberry	Ulmaceae	Indigestion, cough, allergy, colic pain
*Diospyros lotus *L.	Kala malook/Date plum	Ebenaceae	Constipation, indigestion
*Duchesnea indica *(Andr.)	Budi meva/Indian strawberry	Rosaceae	Diarrhea, stomach ulcer
*Grewia optiva *Drum. ex. Burret.	Dhaman/Monkey soap	Tiliaceae	Constipation, antidiabetic, cooling agent
*Juglans regia *L.	Khor/Walnut	Juglandaceae	Brain tonic, asthma, piles, aphrodisiac, dysentery
*Myrsine africana *L.	Khukan/African boxwood	Myrsinaceae	Intestinal worms
*Olea ferruginea *Royle	Kahou/Wild olive	Oleaceae	Joint swelling, appetizer, tonic, piles
*Opuntia dillenii *Haw.	Nakh band/Prickly pear	Cactaceae	Diabetes, ulcer, weakness, diarrhea
*Prunus armeniaca *L.	Harii/Wild apricot	Rosaceae	Constipation, carminative
*Prunus domestica *L.	Lucha/Round plum	Rosaceae	Constipation, stomach inflammation
*Pyrus pashia *L.	Kali Batangi /Black pear tree	Rosaceae	Constipation, cough, fever
*Rosa brunonii *Lindl	Tarni/Himalayan musk rose	Rosaceae	Inflammation, heart problem, constipation
*Rosa moschata *auct.	Gangli gulab/Musk rose	Rosaceae	Stomach inflammation, constipation
*Rubus ellipticus *Smith in Rees.	Akha/Asian wild raspberry	Rosaceae	Diabetes, constipation, nausea, carminative, tonic
*Rubus ulmifolius *Schott in Oken.	Kali bari pluchi/Blackberry	Rosaceae	Inflammation, constipation, diabetes, carminative
*Viburnum grandiflorum* Wall. ex DC.	Guch/Himalayan Viburnum	Caprifoliaceae	Constipation, gastric problem, to purify blood
*Vitis jacquemontii *Parker.	Gidhar dakh/Tree of Heaven	Vitaceae	Constipation
*Vitis parvifolia *Roxb.	Kali bari dakh/Summer grape	Vitaceae	Constipation
*Zanthoxylum armatum *D.C. Prodor.	Timbur/Toothache Tree	Rutaceae	Indigestion, gas trouble, piles

**Table 2:** Free radicals scavenging activity of edible wild fruits.

S. No	Botanical name	%DPPH (WE)	%DPPH (AE)	%OH^−^ (WE)	%OH^−^ (AE)	%H_2_O_2_ (WE)	%H_2_O_2_ (AE)
(1)	*B. lycium*	65.03^i^ ± 7.79	85.22^e^ ± 7.52	49.17^i^ ± 3.21	11.31^q^ ± 1.24	62.02^i^ ± 6.69	72.87^a^ ± 9.87
(2)	*C. australis *	88.60^b^ ± 5.69	54.24^i^ ± 4.69	39.70^l^ ± 4.63	44.74^g^ ± 6.97	54.69^j^ ± 6.31	65.61^b^ ± 8.56
(3)	*D. lotus *	66.31^h^ ± 8.97	94.15^a^ ± 8.75	20.95^n^ ± 3.78	57.68^d^ ± 7.52	40.64^l^ ± 5.59	29.49^j^ ± 3.57
(4)	*D. indica*	79.51^e^ ± 4.32	**94.6**6^**a**^ **± 8.89**	75.40^a^ ± 6.11	**72.5**9^**a**^ **± 6.78**	**83.5**4^**a**^ **± 9.37**	61.63^c^ ± 7.68
(5)	*G. optiva *	34.87^l^ ± 6.25	76.29^g^ ± 7.12	13.15^p^ ± 1.36	63.69^c^ ± 5.59	9.509^n^ ± 1.01	23.41^k^ ± 4.25
(6)	*J. regia *	76.63^f^ ± 4.10	93.35^ab^ ± 7.23	52.77^h^ ± 5.98	14.66^o^ ± 2.56	73.4^d^ ± 8.23	12.33^o^ ± 1.16
(7)	*M. africana *	33.66^m^ ± 5.69	93.86^ab^ ± 6.98	16.13^o^ ± 2.37	38.81^j^ ± 4.65	45.34^k^ ± 3.69	23.04^k^ ± 2.59
(8)	*O. ferruginea *	61.09^j^ ± 8.21	80.43^f^ ± 9.21	62.42^g^ ± 7.76	67.60^b^ ± 7.98	72.34^e^ ± 5.21	63.13^c^ ± 7.58
(9)	*O. dillenii*	71.89^g^ ± 7.83	70.07^h^ ± 5.21	37.82^m^ ± 4.31	39.95^i^ ± 2.69	57.15^h^ ± 4.16	19.91^m^ ± 2.65
(10)	*P. armeniaca *	87.19^c^ ± 7.39	91.42^abcd^ ± 8.90	48.14^j^ ± 4.01	49.70^f^ ± 4.56	77.48^bc^ ± 9.13	**73.0**4^**a**^ **± 9.21**
(11)	*P. domestica *	72.84^g^ ± 7.70	91.61^abcd^ ± 7.98	48.30^ij^ ± 4.36	12.81^p^ ± 1.25	67.43^f^ ± 5.27	55.54^e^ ± 4.56
(12)	*P. pashia *	86.70^c^ ± 9.25	92.50^abc^ ± 9.98	72.32^b^ ± 7.96	51.73^e^ ± 3.58	72.52^d^ ± 8.87	14.96^n^ ± 2.58
(13)	*R. brunonii *	**92.4**7^**a**^ **± 5.31**	91.80^abcd^ ± 10.6	65.92^f^ ± 5.55	35.29^l^ ± 5.58	72.46^d^ ± 7.36	51.51^f^ ± 5.79
(14)	*R. moschata *	62.04^j^ ± 8.36	93.32^ab^ ± 10.9	45.14^k^ ± 5.31	20.88^n^ ± 3.37	67.76^f^ ± 3.47	32.45^i^ ± 2.68
(15)	*R. ellipticus *	91.70^a^ ± 7.69	94.65^a^ ± 9.87	67.63^e^ ± 9.21	42.71^h^ ± 4.59	76.42^c^ ± 9.11	51.83^f^ ± 7.89
(16)	*R. ulmifolius *	67.34^h^ ± 4.33	89.49^cd^ ± 4.36	69.01^d^ ± 4.99	37.31^l^ ± 3.75	48.27^j^ ± 5.67	45.86^g^ ± 6.19
(17)	*V. grandiflorum *	57.17^k^ ± 5.89	84.87^e^ ± 5.30	**75.8**5^**a**^ **± 8.31**	9.840^r^ ± 1.06	41.82^k^ ± 3.67	40.69^h^ ± 5.38
(18)	*V. jacquemontii *	89.03^b^ ± 8.99	89.93^cd^ ± 7.79	65.37^f^ ± 7.77	27.36^m^ ± 4.21	77.70^b^ ± 8.51	2.100^p^ ± 1.00
(19)	*V. parvifolia *	82.22^d^ ± 7.36	91.39^abcd^ ± 8.16	70.71^c^ ± 8.12	11.28^q^ ± 2.56	29.27^m^ ± 4.69	22.71^kl^ ± 3.52
(20)	*Z. armatum *	67.15^h^ ± 7.23	88.91^d^ ± 11.2	71.35^c^ ± 5.10	45.38^g^ ± 5.89	60.88^gh^ ± 7.12	21.64^lm^ ± 2.91

WE: water extract; AE: acetone extract; values are the means of triplicate analysis ± SD. Different letters (a-t) within the columns indicate significant difference at* p*<0.05.

**Table:** (a) Pearson correlation analysis between phenolics and antioxidant properties in water extract

	TPC	TFC	TFlC	DPPH	OH^−^	H_2_O_2_	FRAP	PMA
TPC	1.00							
TFC	.313	1.00						
TFlC	.419	.244	1.00					
DPPH	-.248	-.599(*∗∗*)	-.256	1.00				
OH^−^	-.520(*∗*)	-.465(*∗*)	-.457(*∗*)	.580(*∗∗*)	1.00			
H_2_O_2_	-.146	-.468(*∗*)	-.163	.629(*∗∗*)	.489(*∗*)	1.00		
FRAP	.598(*∗∗*)	.202	.606(*∗∗*)	.154	-.252	.192	1.00	
PMA	.532(*∗*)	.457(*∗*)	.525(*∗*)	-.228	-.361	-.191	.739(*∗∗*)	1.00

*∗* Correlation is significant at the 0.05 level (2-tailed). *∗∗* Correlation is significant at the 0.01 level (2-tailed). TPC: total phenolics content, TFC: total flavonoids content, TFlC: total flavonols content, WE: water extract, AE: acetone extract.

**Table:** (b) Pearson correlation analysis among polyphenolics and antioxidant properties in acetone extract

	TPC	TFC	TFlC	DPPH	OH^−^	H_2_O_2_	FRAP	PMA
TPC	1.00							
TFC	.486(*∗*)	1.00						
TFlC	.352	.472(*∗*)	1.00					
DPPH	.277	-.230	.156	1.00				
OH^−^	-.393	-.264	-.160	-.148	1.00			
H_2_O_2_	.346	.399	.175	-.182	.147	1.00		
FRAP	.546(*∗*)	.576(*∗∗*)	.789(*∗∗*)	.283	-.036	.186	1.00	
PMA	.346	.581(*∗∗*)	.631(*∗∗*)	.200	.033	.131	.627(*∗∗*)	1.00

*∗* Correlation is significant at the 0.05 level (2-tailed). *∗∗* Correlation is significant at the 0.01 level (2-tailed). TPC: total phenolics content, TFC: total flavonoids content, TFlC: total flavonols content, WE: water extract, AE: acetone extract.

## Data Availability

The data used to support the findings of this study are available from the corresponding author upon request.

## References

[1] Hazra B., Sarkar R., Biswas S., Mandal N. (2010). Comparative study of the antioxidant and reactive oxygen species scavenging properties in the extracts of the fruits of terminalia chebula, terminalia belerica and emblica officinalis. *BMC Complementary and Alternative Medicine*.

[2] Abbasi A. M., Shah M. H., Khan M. A. (2015). Wild edible vegetables of lesser himalayas. *Ethnobotanical and Nutraceutical Aspects*.

[3] Abbasi A. M., Shah M. H., Li T., Fu X., Guo X., Liu R. H. (2015). Ethnomedicinal values, phenolic contents and antioxidant properties of wild culinary vegetables. *Journal of Ethnopharmacology*.

[4] Abbasi A. M., Khan M. A., Khan N., Shah M. H. (2013). Ethnobotanical survey of medicinally important wild edible fruits species used by tribal communities of Lesser Himalayas-Pakistan. *Journal of Ethnopharmacology*.

[5] Andradecetto A., Heinrich M. (2011). From the Field into the Lab: Useful Approaches to Selecting Species Based on Local Knowledge. *Frontiers in Pharmacology*.

[6] Turner N. J., Luczaj L. J., Migliorini P. (2011). Edible and tended wild plants, traditional ecological knowledge and Agroecology. *Critical Reviews in Plant Sciences*.

[7] Pieroni A., Nebel S., Santoro R. F., Heinrich M. (2005). Food for two seasons: Culinary uses of non-cultivated local vegetables and mushrooms in a south Italian village. *International Journal of Food Sciences and Nutrition*.

[8] Heinrich M. (2000). Ethnobotany and its role in drug development. *Phytotherapy Research*.

[9] Kaur C., Kapoor H. C. (2001). Antioxidants in fruits and vegetables—the millennium's health. *International Journal of Food Science & Technology*.

[10] Fang Y.-Z., Yang S., Wu G. (2002). Free radicals, antioxidants, and nutrition. *Nutrition Journal *.

[11] Wargovich M. J. (2000). Anticancer properties of fruits and vegetables. *HortScience*.

[12] Prior R. L., Cao G. (2000). Antioxidant phytochemicals in fruits and vegetables: Diet and health implications. *HortScience*.

[13] Karakaya S., Kavas A. (1999). Antimutagenic activities of some foods. *Journal of the Science of Food & Agriculture*.

[14] Cao S.-G., Sim K.-Y., Pereira J., Goh S.-H. (1998). Coumarins from Calophyllum teysmannii (Guttiferae). *Phytochemistry*.

[15] Newman D. J., Cragg G. M. (2007). Natural products as sources of new drugs over the last 25 years. *Journal of Natural Products*.

[16] Khan M. P. Z., Ahmad M. (2015). Traditional preference of Wild Edible Fruits (WEFs) for digestive disorders (DDs) among the indigenous communities of Swat Valley-Pakistan. *Journal of Ethnopharmacology*.

[17] Pardo-de-Santayana M., Tardío J., Blanco E. (2007). Traditional knowledge of wild edible plants used in the northwest of the Iberian Peninsula (Spain and Portugal): a comparative study. *Journal of Ethnobiology and Ethnomedicine*.

[18] Abuye C., Urga K., Knapp H. (2003). A compositional study of Moringa stenopetala leaves. *East African Medical Journal*.

[19] Ji L., Wu J., Gao W., Wei J., Yang J., Guo C. (2011). Antioxidant capacity of different fractions of vegetables and correlation with the contents of ascorbic acid, phenolics, and flavonoids. *Journal of Food Science*.

[20] Chen Y., Chen G., Fu X., Liu R.-H. (2015). Phytochemical profiles and antioxidant activity of different varieties of *Adinandra* tea (Adinandra Jack). *Journal of Agricultural and Food Chemistry*.

[21] Chen Y., Ma X., Fu X., Yan R. (2017). Phytochemical content, cellular antioxidant activity and antiproliferative activity of: Adinandra nitida tea (Shiyacha) infusion subjected to in vitro gastrointestinal digestion. *RSC Advances*.

[22] Kumaran A., Joel Karunakaran R. (2006). Antioxidant and free radical scavenging activity of an aqueous extract of *Coleus aromaticus*. *Food Chemistry*.

[23] Chen Y., Zhang R., Liu C., Zheng X., Liu B. (2016). Enhancing antioxidant activity and antiproliferation of wheat bran through steam flash explosion. *Journal of Food Science and Technology*.

[24] Yu W., Zhao Y., Shu B. (2004). The radical scavenging activities of radix puerariae isoflavonoids: a chemiluminescence study. *Food Chemistry*.

[25] Aiyegoro O. A., Okoh A. I. (2010). Preliminary phytochemical screening and In vitro antioxidant activities of the aqueous extract of Helichrysum longifolium DC. *BMC Complementary and Alternative Medicine*.

[26] Prieto P., Pineda M., Aguilar M. (1999). Spectrophotometric quantitation of antioxidant capacity through the formation of a phosphomolybdenum complex: specific application to the determination of vitamin E. *Analytical Biochemistry*.

[27] Shrestha P. M., Dhillion S. S. (2006). Diversity and traditional knowledge concerning wild food species in a locally managed forest in Nepal. *Agroforestry Systems*.

[28] Ajesh T. P., Naseef S. A. A., Kumuthakalavalli R. (2012). Preliminary study on the utilization of wild vegetables by muthuvan tribes of idukki district of kerala, india. *International Journal of Applied Biology & Pharmaceutical Technology*.

[29] Khan M. A., Khan M. A., Hussain M., Mujtaba G. (2010). An ethnobotanical inventory of himalayan region poonch valley azad kashmir (Pakistan). *Ethnobotany Research and Applications *.

[30] Khan N., Abbasi A. M., Dastagir G. (2014). Ethnobotanical and antimicrobial study of some selected medicinal plants used in *Khyber Pakhtunkhwa* (KPK) as a potential source to cure infectious diseases. *BMC Complementary and Alternative Medicine*.

[31] Juan M. E., González-Pons E., Planas J. M. (2010). Multidrug resistance proteins restrain the intestinal absorption of trans-resveratrol in rats. *Journal of Nutrition*.

[32] Temple N. J. (2000). Antioxidants and disease: More questions than answers. *Nutrition Research*.

[33] Liu M., Li X. Q., Weber C., Lee C. Y., Brown J., Liu R. H. (2002). Antioxidant and antiproliferative activities of raspberries. *Journal of Agricultural and Food Chemistry*.

[34] Wolfe K. L., Liu R. H. (2008). Structure-activity relationships of flavonoids in the cellular antioxidant activity assay. *Journal of Agricultural and Food Chemistry*.

[35] Bravo L. (1998). Polyphenols: chemistry, dietary sources, metabolism, and nutritional significance. *Nutrition Reviews*.

[36] Aliyu A. B., Musa A. M., Oshanimi J. A., Ibrahim H. A., Oyewale A. O. (2008). Phytochemical analyses and mineral elements composition of some medicinal plants of northern nigeria. *Nigerian Journal of Pharmaceutical Sciences*.

[37] Sood P., Modgil R., Sood M. (2010). Physico-chemical and nutritional evaluation of indigenous wild fruit Kasmal, Berberis lycium Royle. *Indian Journal of Natural Products and Resources (IJNPR)*.

[38] Singh T. P., Singh O. M. (2011). Phytochemical and pharmacological profile of Zanthoxylum armatum DC. - An overview. *Indian Journal of Natural Products & Resources*.

[39] Batool F., Sabir S. M., Rocha J. B. T., Shah A. H., Saify Z. S., Ahmed S. D. (2010). Evaluation of antioxidant and free radical scavenging activities of fruit extract from Zanthoxylum alatum: A commonly used spice from Pakistan. *Pakistan Journal of Botany*.

[40] Seal T. (2012). Antioxidant activity of some wild edible plants of meghalaya state of india: A comparison using two solvent extraction systems. *International Journal of Nutrition & Metabolism*.

[41] Fu L., Xu B.-T., Xu X.-R., Qin X.-S., Gan R.-Y., Li H.-B. (2010). Antioxidant capacities and total phenolic contents of 56 wild fruits from South China. *Molecules*.

[42] Lamien-Meda A., Lamien C., Compaoré M. (2008). Polyphenol content and antioxidant activity of fourteen wild edible fruits from Burkina Faso. *Molecules*.

[43] Karuppusamy G. M. S., Rajasekaran K. M. (2011). Antioxidant activity of selected lesser known edible fruits from western ghats of india. *Molecular & Cellular Endocrinology*.

[44] Sun J., Chu Y.-F., Wu X., Liu R. H. (2002). Antioxidant and antiproliferative activities of common fruits. *Journal of Agricultural and Food Chemistry*.

[45] Escarpa A., González M. C. (2001). Approach to the content of total extractable phenolic compounds from different food samples by comparison of chromatographic and spectrophotometric methods. *Analytica Chimica Acta*.

[46] Lemarchand L. (2002). Cancer preventive effects of flavonoids—a review. *Biomedicine & Pharmacotherapy*.

[47] Yao L. H., Jiang Y. M., Shi J. (2004). Flavonoids in food and their health benefits. *Plant Foods for Human Nutrition*.

[48] Yang J., Liu R. H., Halim L. (2009). Antioxidant and antiproliferative activities of common edible nut seeds. *LWT - Food Science and Technology*.

[49] Medina-Remón A., Barrionuevo-González A., Zamora-Ros R. (2009). Rapid Folin-Ciocalteu method using microtiter 96-well plate cartridges for solid phase extraction to assess urinary total phenolic compounds, as a biomarker of total polyphenols intake. *Analytica Chimica Acta*.

[50] Lamuela-Raventós R. M., Singleton V. L., Orthofer R. (1999). *Analysis of Total Phenols and Other Oxidation Substrates and Antioxidants by Means of Folin-Ciocalteu Reagent*.

[51] Maria R., Fernandes F., Alves R., Debrito E. (2009). Free radical-scavenging behaviour of some north-east Brazilian fruits in a DPPH system. *Food Chemistry*.

[52] Tenore G. C., Novellino E., Basile A. (2012). Nutraceutical potential and antioxidant benefits of red pitaya (Hylocereus polyrhizus) extracts. *Journal of Functional Foods*.

[53] Chen Y., Shen Y., Fu X., Abbasi A. M., Yan R. (2017). Stir-frying treatments affect the phenolics profiles and cellular antioxidant activity of Adinandra nitida tea (Shiyacha) in daily tea model. *International Journal of Food Science & Technology*.

[54] Huang D., Ou B., Prior R. L. (2005). The chemistry behind antioxidant capacity assays. *Journal of Agricultural and Food Chemistry*.

[55] Ma X., Wu H., Liu L. (2011). Polyphenolic compounds and antioxidant properties in mango fruits. *Scientia Horticulturae*.

[56] Peng Q., Wei Z., Lau B. H. S. (2000). Pycnogenol inhibits tumor necrosis factor-alpha-induced nuclear factor kappa B activation and adhesion molecule expression in human vascular endothelial cells. *Cellular and Molecular Life Sciences*.

[57] Aparadh V. T., Naik V. V., Karadge B. A. (2012). Antioxidative properties (TPC, DPPH, FRAP, Metal Chelating Ability, Reducing Power and TAC) within some Cleome species. *Annali di Botanica*.

[58] Motamed S. M., Naghibi F. (2010). Antioxidant activity of some edible plants of the Turkmen Sahra region in northern Iran. *Food Chemistry*.

[59] Balaban R. S., Nemoto S., Finkel T. (2005). Mitochondria, oxidants, and aging. *Cell*.

[60] Shi H., Hudson L. G., Ding W. (2004). Arsenite causes DNA damage in keratinocytes via generation of hydroxyl radicals. *Chemical Research in Toxicology*.

[61] Alam M. N., Bristi N. J., Rafiquzzaman M. (2013). Review on *in vivo* and *in vitro* methods evaluation of antioxidant activity. *Saudi Pharmaceutical Journal*.

[62] Miller W. R., Benefield R. G., Tonigan J. S. (1993). Enhancing motivation for change in problem drinking: A controlled comparison of two therapist styles. *Journal of Consulting and Clinical Psychology*.

[63] Miladi S., Damak M. (2008). In vitro antioxidant activities of aloe vera leaf skin extracts. *Journal De La Société Chimique De Tunisie*.

[64] Benzie I. F. F., Strain J. J. (1999). Ferric reducing/antioxidant power assay: direct measure of total antioxidant activity of biological fluids and modified version for simultaneous measurement of total antioxidant power and ascorbic acid concentration. *Methods in Enzymology*.

[65] Meyers K. J., Watkins C. B., Pritts M. P., Liu R. H. (2003). Antioxidant and antiproliferative activities of strawberries. *Journal of Agricultural and Food Chemistry*.

[66] Jan S., Khan M. R., Rashid U., Bokhari J. (2013). Assessment of antioxidant potential, total phenolics and flavonoids of different solvent fractions of monotheca buxifolia fruit. *Osong Public Health and Research Perspectives*.

